# False Elevation of Parathyroid Hormone in a Patient With Lung Metastasis of Rectal Cancer After Immunotherapy: A Case Report and Literature Review

**DOI:** 10.1155/crii/8400162

**Published:** 2026-03-09

**Authors:** Chang-Sheng Xia, Lingli Zhou, Chendi Jing, Chunhong Fan, Yejiao Hong, Zhi-Hong Yue, Leili Gao, Fang Ren

**Affiliations:** ^1^ Department of Clinical Laboratory, Peking University People’s Hospital, Beijing, 100044, China, pku.edu.cn; ^2^ Department of Endocrinology and Metabolism, Peking University People’s Hospital, Beijing, 100044, China, pku.edu.cn; ^3^ Department of Endocrinology and Metabolism, People’s Hospital of Jinchang, Jinchang, 737199, Gansu Province, China

**Keywords:** heterophile antibodies, human anti-mouse antibodies, immunoassay interference, parathyroid hormone

## Abstract

**Background:**

Immunoassays are commonly used in clinical laboratories to measure a variety of analytes, including hormones and tumor markers. Interference caused by rheumatoid factor (RF), heterophile antibodies, and human anti‐animal antibodies (HAAA) has been reported but is rarely identified in daily practice. Here, we report a case of falsely elevated parathyroid hormone (PTH) due to immunoassay interference and review the literature.

**Case Presentation:**

A 57‐year‐old man who recovered well from lung metastasis of rectal cancer treated with bevacizumab and sintilimab for 1 year, presented to Peking University People’s Hospital with persistently high PTH levels (>1200 ng/L) measured by a Roche Elecsys assay. He had hypoadrenocorticism induced by anti‐programmed cell death 1 (PD‐1), normal renal function, normal total calcium level, and normal 25‐OH vitamin D concentration. The Beckman Coulter UniCel DxI 800 and Siemens Immulite 2000 platforms measured PTH levels of 18.3 ng/L and 8.7 ng/L, respectively. After the patient’s serum was treated with polyethylene glycol (PEG) precipitation or mouse serum, the PTH levels determined by the Roche immunoassay decreased to 56.5 ng/L and 265.3 ng/L, respectively.

**Conclusion:**

Interference due to human anti‐mouse antibodies (HAMA) could be the cause of falsely elevated PTH in the patient. Physicians should realize that immunoassay interference can lead to false results and closely communicate with the laboratory to avoid misdiagnosis and inappropriate therapies.

## 1. Introduction

Parathyroid hormone (PTH) is routinely measured in clinical laboratories using different automated platforms that use a chemiluminescence immunoassay to diagnose and monitor hyperparathyroidism and hypoparathyroidism, including platforms from Roche Diagnostics, Beckman Coulter, Abbott Diagnostics, Siemens Healthcare Diagnostics, and DiaSorin [1–3]. Immunoassays for detecting PTH use a “sandwich” technique with a capture antibody (solid phase) targeting the N‐terminal fragment and a signal antibody targeting the C‐terminal fragment. Despite their specificity, the immunoassays are inherently vulnerable to interference by immunoglobulin‐related substances, including rheumatoid factor (RF), heterophile antibodies, human anti‐animal antibodies (HAAA), and anti‐analyte antibodies. These interfering substances, capable of bridging the capture and signal antibodies, can cause falsely elevated results, leading to misdiagnosis and unnecessary therapies. However, interference is difficult to identify in routine practice, and the prevalence may be underestimated.

Here, we report a case of persistently high levels of PTH that finally turned out to be caused by immunoassay interference due to human anti‐mouse antibodies (HAMA) in a man with hypoadrenocorticism, normal renal function, normal total calcium level, and normal 25‐OH vitamin D level.

## 2. Case Presentation

A 57‐year‐old Chinese man presented to Peking University People’s Hospital with persistently high PTH levels on February 16, 2023. He had been diagnosed with hypoadrenocorticism induced by anti‐programmed cell death 1 (PD‐1) and received oral prednisone (5 mg in the morning, 2.5 mg in the evening, and daily) and had been treated with sintilimab combined with bevacizumab for lung metastasis of rectal cancer from February 2021 to February 2022. He had recovered well in a tumor treatment center (Beijing Chaoyang Huanxing Cancer Hospital). Using the Roche Elecsys assay (Roche Diagnostics, Mannheim, Germany) on the Roche Cobas e601 immunoassay analyzer (reference interval, 15–65 ng/L) at the tumor treatment center, his first PTH level was 1366 ng/L (January 10, 2023), and his second PTH level was 1297 ng/L (February 11, 2023). A series of examinations of the high PTH levels was performed before referral to the Metabolism and Endocrinology Department at our tertiary hospital in Beijing, China. His serum PTH was measured using the same platform at our hospital and found to be 1542 ng/L (February 16, 2023), higher than previous measurements. His other laboratory results, including serum levels of total calcium, phosphorus, creatinine, 25‐OH vitamin D, bone alkaline phosphatase (BAP), and tartrate‐resistant acid phosphatase (TAP), as well as liver function tests, thyroid function tests, and indicators of glucose and lipid metabolism, were normal except for low levels of albumin, total protein, and creatinine (Table 1). Ultrasound imaging revealed that both pairs of parathyroid glands were normal. Additionally, two small nodules were detected in the right lobe of the thyroid gland, the largest measuring ~0.5 cm× 0.3 cm, with normal vascularization. Technetium‐99m methoxyisobutylisonitrile (99Tc‐MIBI) dual‐phase planar imaging and single‐photon emission computed tomography/computed tomography (SPECT/CT) revealed normal 99Tc uptake in both thyroid lobes and both pairs of parathyroid glands at 15 min, 2, and 9 h postinjection. The patient had no history of fractures, malabsorption, kidney stones, or renal insufficiency. His physical examination revealed no abnormalities.

**Table 1:** The patient’s other laboratory results.

Test	Result	Reference interval
Liver function^a^		
ALT (U/L)	20	9–50
AST (U/L)	31	15–40
ALP (U/L)	45	45–125
GGT (U/L)	12	10–60
LDH (U/L)	186	120–250
Total protein (g/L)	58.6	65–85
Albumin (g/L)	33.7	40–55
TBIL (µmol/L)	15.1	3.4–17.1
Renal function^a^		
Urea (mmol/L)	3.7	3.1–8.0
Creatinine (µmol/L)	51.0	57–97
Uric acid (µmol/L)	270	208–428
Cystatin C (mg/L)	0.97	0 59–1.53
Glucose and lipid metabolism^a^		
Glucose (mmol/L)	4.53	3.89–6.11
Total cholesterol (mmol/L)	3.57	0–5.18
Triglyceride (mmol/L)	0.79	0–1.7
Inorganic ions^a^		
Total calcium (mmol/L)	2.29	2.11–2.52
Phosphorus (mmol/L)	1.31	0.85 −1.51
Ferric ion (µmol/L)	12.1	10.6–36.7
Magnesium (mmol/L)	0.76	0.75–1.02
Bone metabolism		
25‐OH vitamin D (nmol/L)^b^	95.75	75–250 (sufficient)
BAP (µg/L)^c^	11.24	8–16.6
TAP (U/L)^c^	3.27	2.18–3.94
Thyroid function^b^		
TG (ng/mL)	48.71	1.4–78
T3 (nmol/L)	2.52	1.3–3.1
T4 (nmol/L)	103.70	69–141
fT3 (pmol/L)	5.77	4.6–7.8
fT4 (pmol/L)	15.06	12–22
TSH (μIU/mL)	1.01	0.27–4.20
Thyroid uptake (TBI)	1.07	0.8–1.3
TRAb (IU/L)	0.99	0–1.22
TGAb (IU/mL)	14.83	0–115
TPOAb (IU/mL)	5.64	0–34

*Note:* fT3, free triiodothyronine; fT4, free tetraiodothyronine; T3, 3, 5, 3’‐triiodothyronine; T4, thyroxine, 3, 5, 3’‐tetraiodothyronine.

Abbreviations: ALP, alkaline phosphatase; ALT, alanine aminotransferase; AST, aspartate aminotransferase; BAP, bone alkaline phosphatase; GGT, gamma‐glutamyl transferase; LDH, lactate dehydrogenase; TAP, tartrate‐resistant acid phosphatase; TBI, thyroid‐binding index; TBIL, total bilirubin; TG, thyroglobulin; TGAb, thyroglobulin autoantibodies; TPOAb, thyroid peroxidase autoantibodies; TRAb, thyrotropin receptor autoantibodies; TSH, thyroid stimulating hormone.

^a^Conducted using Beijing Strong Biotechnologies, Inc. reagents on the Hitachi Labospect 008 AS automatic analyzer.

^b^Performed using a Roche Cobas e601 immunoassay analyzer and Roche reagents.

^c^Measured using enzyme‐linked immunosorbent assay reagents (Phicon Biotechnology Co., Ltd, Guangzhou) on the Bio‐Rad automatic enzyme immunoassay system Evolis.

High PTH concentrations are caused mainly by hyperparathyroidism, renal insufficiency, and vitamin D insufficiency or deficiency. Given the discordance between the patient’s high PTH levels and clinically unremarkable presentation, immunoassay interference was raised as the possible cause of the apparent erroneously elevated PTH. We performed blood sampling and tested PTH again on March 16, 2023. Briefly, 200 μL of the patient serum was treated with 200 μL of 25% polyethylene glycol (PEG) 4000 precipitation. After centrifugation, PTH was detected in the supernatant using the Roche Elecsys assay, and the result was corrected for dilution times [4]. Neat serum from the patient was also used to measure PTH. After adding the PEG precipitation, the serum PTH levels decreased from 1221 to 56.5 ng/L (percent change −95.4%, more than 20%; Table 2). If there was no interference, an acceptable percent change should be between −20% and + 20% due to the dilution effect [5]. This confirmed our hypothesis that immunoassay interference caused falsely elevated PTH levels. Moreover, control serum from a patient with hyperparathyroidism was used to detect PTH levels before and after PEG treatment, and the results showed no significant difference (percent change 5.3%). This proved that PEG pretreatment itself did not interfere in the measurement of PTH‐positive samples.

**Table 2:** Experiments for identifying false elevation of PTH.

Treatment	PTH (ng/L)	Percent change^c^
PEG 4000 treatment testing^a^		
Patient serum		
Before treatment	1221	NA
After treatment	56.5	−95.4%
Control serum^b^		
Before treatment	734.6	NA
After treatment	773.2	5.3%
Dilution testing^a^		
Neat	1221	NA
1:6.67 dilution	1295	6.1%
1:10 dilution	1081	−11.5%
1:20 dilution	1349	10.5%
Different platform testing		
Beckman UniCel DxI 800 assay	18.3	NA
Siemens Immulite 2000 assay	8.7	NA
Mouse serum treatment testing^a^		
Patient serum		
Before treatment	1221	NA
After treatment	265.3	−78.3%
Control serum^b^		
Before treatment	309.2	NA
After treatment	303.0	−2.0%
Mouse serum	1.4	NA

Abbreviations: NA, not available; PEG, polyethylene glycol; PTH, parathyroid hormone.

^a^Roche Elecsys assay.

^b^Two remaining serum specimens from two patients with hyperparathyroidism.

^c^Percent change = (post − pre)/pre × 100%.

To identify the exact interference factor in the patient presenting for PTH testing, we tested for RF using Beckman immunoturbidimetric reagent on the Beckman biochemical analyzer AU5800 and found that it was negative. Subsequently, serum dilution testing was performed with universal diluent (Roche Diagnostics), and the percent changes were within ±20%, showing linearity (Table 2).

In general, serum dilution tests showing a linear pattern exclude interference [6]. However, our results suggest that serum dilution tests to determine the presence or absence of interference are unreliable. We sent the patient‐splitting serum specimens to two other hospitals for PTH detection with different platforms. A Beckman Coulter UniCel DxI 800 found PTH to be 18.3 ng/L (reference interval, 12–88 ng/L). A Siemens Immulite 2000 found the level of PTH to be slightly low (8.7 ng/L; reference interval, 12–65 ng/L). The three reagents for PTH testing were all based on a second‐generation two‐step “sandwich” immunoassay. However, the interference only occurred in the Roche immunoassay. The Roche Elecsys assay used two mouse monoclonal antibodies, whereas the Beckman Coulter and Siemens assays used a mouse monoclonal antibody and goat polyclonal antibodies. Thus, high PTH levels with the Roche Elecsys assay may be due to interference by HAMA. To confirm that interference was due to HAMA, the patient serum was incubated with mouse serum (30 µL of patient serum + 170 µL of mouse serum) for 30 min at room temperature to neutralize any HAMA that were present. After centrifugation, the concentration of PTH in the supernatant was measured. The patient’s serum PTH levels determined by the Roche Elecsys assay decreased from 1221 to 265.3 ng/L (Table 2), which is still higher than the upper limit of the reference interval. The percent change in PTH levels was −78.3%, confirming interference by HAMA in the patient’s serum. We also utilized PTH‐positive serum, readily available in our laboratory, as a control. Our analysis revealed no significant differences in PTH levels before and after treatment with mouse serum (percent change −2.0%). The PTH level in mouse serum was found to be 1.4 ng/L (Table 2).

Taken together, the findings indicate that the PTH levels in this patient were falsely elevated due to interference by HAMA. The patient did not receive relative treatment for high PTH concentrations and was maintained on oral prednisone for hypoadrenocorticism, with continuously improving outcomes. The patient was followed up annually after the initial visit to our hospital, with a total of two follow‐up visits. No symptoms of hyperparathyroidism, such as kidney stones or bone pain, were observed during this period.

## 3. Discussion

Immunoassay interference occurs in serum PTH testing due to heterophile antibodies, HAMA, and RF, among other factors. However, heterophile antibodies are often confused with HAAA. A commercial reagent called heterophilic blocking reagent (HBR), or heterophile‐blocking tube (HBT) (Scantibodies Laboratory), misleads users. The reagent can block antibody‐related interference, including heterophile antibodies, HAAA, and RF. When this reagent is used and the interference blocked, the researchers usually state that the interference was caused by heterophile antibodies. The interference may actually be due to heterophile antibodies, HAAA, RF, or other factors. As early as 1999, Kaplan and Levinson [7] called for the correct definition of these antibodies and proposed a simple nomenclature. Briefly, heterophile antibodies are weakly multispecific antibodies and are considered to occur naturally. In contrast, HAAA, such as HAMA, are monospecific high‐affinity antibodies that can appear after exposure to animals or animal products, or without any identified cause [7–9].

Some cases of false PTH elevation were reported to be due to immunoassay interference. Cavalier et al. [5] showed that, of 63 suspicious samples with high PTH levels tested by the DiaSorin Liaison assay and treated with HBT and RF‐absorbent, 40% were heterophile antibody‐positive and 14% were confirmed to be interfered with by RF. They referred to heterophile antibodies as HAMA. Calabrò et al. [6] reported that repeatedly high PTH levels when tested with Beckman Coulter UniCel were caused by heterophile interference and interference related to alkaline phosphatase by using HBR and alkaline phosphatase mutein. Zanchetta et al. [4] reported a case of asymptomatically elevated PTH levels due to immunoassay interference by heterophile antibodies as confirmed by sample dilution with Roche and Siemens assays and PEG precipitation. Kim et al. [10] presented three cases of abnormally high PTH levels measured by the Roche assay after natural killer cell therapy. They attributed the interference to heterophile antibodies for HBT blocking. van der Doelen et al. [11] reported an interesting case of primary hyperparathyroidism that became extraordinary when PTH levels remained markedly elevated after surgical therapy. The serum PTH levels were high with the Abbott Architect assay but not with the Roche Elecsys assay, Beckman Access assay, DiaSorin Liaison assay, Siemens ADVIA Centaur assay, or Siemens Immunite assay. The authors attributed the interference to heterophile antibodies by using sample dilution, HBT blocking, goat serum treatment, and PEG precipitation. In addition, Cetani et al. [12] described a case of unexplained elevations in serum PTH levels measured by the DiaSorin Liaison second and third‐generation assays caused by macro‐PTH based on PEG treatment and sample dilution.

Similar to our study, two other cases demonstrated that extremely high PTH levels determined by the Roche Elecsys assay were due to interference by HAMA, and this interference did not occur with other tests, such as thyroid‐stimulating hormone (TSH) and calcitonin, that are also measured using sandwich assays with mouse antibodies [13, 14]. Levin et al. [13] proved the existence of HAMA using HBR and a mouse IgG–agarose affinity column. Laudes et al. [14] confirmed that the immunoassay interference was due to the presence of HAMA using PEG precipitation, HBR blocking, Euroimmun assay, and human anti‐mouse IgG antibody testing. In our study, in addition to PEG treatment and using two different assays to identify the existence of immunoassay interference, the patient’s serum was treated with mouse serum to confirm the existence of HAMA, and we used PTH‐positive serum as a control. Our results demonstrated a significant reduction in the PTH concentration after treatment of the patient’s serum with mouse serum, though the patient’s PTH level remained significantly higher than the upper limit of the normal range. Unfortunately, due to insufficient residual patient serum, a second round of testing—using either HBT or a HAMA‐specific ELISA kit—could not be performed to obtain a definitive result.

The presence of HAMA is sometimes due to keeping mice, engaging in occupations that involve long‐term contact with mice, or receiving therapy with mouse‐derived monoclonal antibodies [13]. Sometimes, the exact cause cannot be identified [14]. The patient in our case report has never kept pets or been in long‐term contact with animals. He received bevacizumab and sintilimab to treat lung metastasis of rectal cancer. Bevacizumab is a human–mouse chimeric monoclonal antibody against vascular endothelial growth factor (VEGF), whereas sintilimab is a fully human IgG4 monoclonal antibody targeting PD‐1 [15, 16]. The Fab fragment of bevacizumab that binds VEGF is derived from mice [17, 18] and is highly suspected of causing the HAMA development in the patient.

Like our study, previous treatment with mouse‐derived monoclonal antibodies (Orthoclone OKT3) may have resulted in the development of HAMA in the patient of Levin et al.’s [13] case report. It is a pity that Laudes et al. [14] did not discuss this question in their study. Interestingly, we found that only the Roche Elecsys PTH assay was affected by the interference by HAMA, whereas the Roche Elecsys TSH assay yielded a normal result. This specificity suggests that the HAMA is likely an anti‐idiotypic antibody targeting an idiotope. Its Fab fragment appears to have high affinity for the Fab fragments of two mouse‐derived antibodies within the Roche PTH assay reagents, causing interference via a Fab–Fab bridging mechanism. This ambiguity is inherent to the bridging mechanism, as it remains unclear which Fab (HAMA’s or the reagent’s) acts as the primary binding site.

The differential outcomes between PEG precipitation (which normalized the PTH value) and mouse serum treatment (which only partially reduced it) further support this hypothesis. While PEG nonspecifically precipitates all large proteins, mouse serum may be insufficient to completely neutralize a high‐titer, high‐affinity, idiotype‐specific antibody. Our data indicate that the patient’s HAMA was an anti‐idiotypic antibody with specificity for partial mouse antibody Fab fragments, explaining the persistent interference despite neutralization with a 5.67‐fold excess of mouse serum. We acknowledge that while HAMA is the primary interferent identified, minor contributions from other factors cannot be entirely ruled out.

This Fab–Fab bridge between the mouse‐derived capture antibody and mouse‐derived detection antibody mimics the antibody–antigen–antibody complex and produces a false‐positive result. Manufacturers should be aware of this potential problem and can add blocking agents to their immunoassay reagents to minimize the effect of HAAA. The blocking agents may consist of nonimmune animal serum or animal‐specific antibodies.

To our knowledge, this is the first case report suggesting that the targeted drug bevacizumab can probably induce HAMA capable of interfering with Roche PTH measurements. Physicians should pay more attention to possible interference in PTH detection from the application of bevacizumab. Further studies with larger sample sizes are warranted to confirm and generalize these findings.

In conclusion, we report a case of falsely elevated PTH due to immunoassay interference by HAMA. The case demonstrates the importance of good communication between clinicians and the laboratory when a laboratory result does not match the clinical manifestations. To avoid misdiagnosis and inappropriate treatments, clinicians should keep in mind that immunoassay interference can lead to false results.

## Author Contributions

Chang‐Sheng Xia and Lingli Zhou designed the study. Chendi Jing and Chunhong Fan performed the PTH tests. Chang‐Sheng Xia, Lingli Zhou, Chendi Jing, Chunhong Fan, Yejiao Hong, Zhi‐Hong Yue, Leili Gao, and Fang Ren drafted the initial manuscript. Chang‐Sheng Xia and Lingli Zhou revised the manuscript.

## Funding

We thank the Doctoral Fund of Ministry of Education of China (Grant 20120001120053) for funding this study.

## Disclosure

All authors approved the final draft submitted.

## Ethics Statement

Written informed consents were obtained from the patients for publication of this case report. This study was reviewed and granted exemption by the Ethics Committee of Peking University People’s Hospital.

## Conflicts of Interest

The authors declare no conflicts of interest.

## Data Availability

The data used to support the findings of this study are included within the article.
